# 4,8-Sphingadienine and 4-hydroxy-8-sphingenine activate ceramide production in the skin

**DOI:** 10.1186/1476-511X-11-108

**Published:** 2012-08-31

**Authors:** Yoshiyuki Shirakura, Kanako Kikuchi, Kenji Matsumura, Katsuyuki Mukai, Susumu Mitsutake, Yasuyuki Igarashi

**Affiliations:** 1 R&D Center, UNITIKA Ltd, 23 Kozakura, Uji-shi, Kyoto, 611-0021, Japan; 2 The Graduate School of Life Science, Faculty of Advanced Life Science, Hokkaido University, Nishi 11, Kita 21, Kita-ku, Sapporo, 001-0021, Japan

**Keywords:** 4,8-sphingadienine, 4-hydroxy-8-sphingenine, Ceramide synthesis

## Abstract

**Background:**

Ingestion of glucosylceramide improves transepidermal water loss (TEWL) from the skin, but the underlying mechanism by which a small amount of dietary glucosylceramide can vastly improve skin conditions remains unclear. In a previous report, glucosylceramides were shown to be digested to sphingoids, which were shown to be absorbed through the intestinal epithelium. Based on these observations, we hypothesized that sphingoids are the key molecules facilitating endogenous ceramide production. In this study, we assessed the effect of 4,8-sphingadienine (d18:2) and 4-hydroxy-8-sphingenine (t18:1), derived from konjac glucosylceramide, on stimulating ceramide production.

**Methods:**

Konjac glucosylceramide acidolysis was performed using hydrochloric acid; the resulting d18:2 and t18:1 were fractionated by column chromatography. Real-time quantitative RT-PCR was performed to assess the effect of d18:2 and t18:1 on gene expression in normal human epidermal keratinocytes, while their effect on the nuclear receptor, peroxisome proliferator-activated receptor (PPAR)γ, was measured using a receptor-cofactor assay system. The effect of d18:2 and t18:1 on stimulating ceramide production was evaluated using HPTLC analysis in a 3-dimensional human skin model.

**Results:**

We noted the upregulation of genes related to *de novo* ceramide synthesis as well as of those encoding the elongases of very long-chain fatty acids by d18:2 and t18:1, but not by glucosylceramide and 4-sphingenine. Both these sphingoids also facilitated the expression of PPARβ/δ and PPARγ; moreover, they also demonstrated ligand activity for PPARγ. These results indicated that d18:2 and t18:1 promote the differentiation of keratinocytes. Analysis of the lipids within the 3-dimensional human skin model indicated that treatment with d18:2 and t18:1 not only upregulated gene expression but also increased ceramide production.

**Conclusions:**

The sphingoids d18:2 and t18:1 activated genes related to *de novo* ceramide synthesis and increased ceramide production, whereas glucosylceramide and 4-sphingenine could not. These results suggest that the effect of dietary glucosylceramides on the skin is mediated by d18:2 and t18:1.

## Background

**Figure 1:**
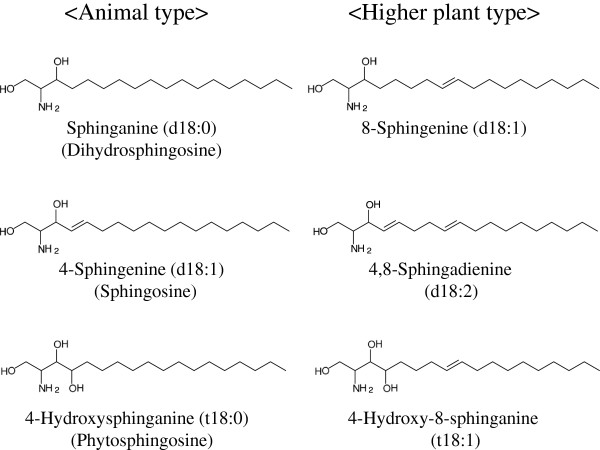
**Structure of animal- and higher plant-type sphingoids.** The consensus structure of sphingoids from animals and higher plants. The higher plant-type is characterized by a double bond between C8 and C9.

Ceramide is a sphingolipid that is composed of a long-chain sphingoid base with 2-amide groups linked to a fatty acid [1,2]. Because of the various combinations of diverse sphingoids and fatty acids, ceramide is a generic name used for more than 10 such molecular species in humans [3]. Ceramides are found not only in animals but also in plants and fungi. Each ceramide has characteristic structures. Ceramides derived from plants are mostly glucosylated (glucosylceramide), while the sphingoid residue mainly has a double bond between C8 and C9 [4]. Fig. 1 shows the typical sphingoid structures derived from animals and from higher plants. Sphingoid structures from fungi share features with those from plants, but, in addition, are methylated at C9 [5,6]. 

Animal ceramides are present not only in the plasma membranes of cells but also in the extracellular matrix of the stratum corneum, where the ceramide content among the intracellular lipids of the stratum corneum reaches approximately 50%. Ceramides and their metabolites form the multilamellar permeability barrier [7]; thus, a reduction in ceramides causes some skin disorders such as atopic dermatitis [8]. Ceramides are also important in signal transduction. For example, phosphorylated ceramide and its metabolites act as an intracellular signaling factor for inducing apoptosis [9].

Uchiyama *et al*. reported that dietary glucosylceramides derived from the konjac tuber reduce transepidermal water loss (TEWL) from the skin [10]. The mechanism by which a small amount of dietary glucosylceramides can improve skin conditions remains unclear. In this study, we attempted to assess the relationship between dietary glucosylceramides and ceramide production in the skin.

A previous study reported that glucosylceramides are digested to sphingoids, which are then absorbed though the intestinal epithelium [11]. Under the hypothesis that skin condition improvement may be mediated by the signaling function of sphingoids, we assessed the ability of sphingoids to induce gene expression in normal human epidermal keratinocytes (NHEK), as well as their nuclear receptor binding activities. The effect of ceramide production was evaluated with the TEST SKIN^TM^ LSE-high, a 3-dimensional cultured human skin model.

## Methods

### Materials

Glucosylceramide derived from konjac tuber was purchased from Nagara Science (Gifu, Japan). 4-Sphingenine and PCR primers were purchased from Sigma Aldrich Japan (Tokyo, Japan). Isogen was purchased from Nippon Gene (Tokyo, Japan). Reverse transcriptase, SYBR-Premix Ex-taq were purchased from TaKaRa Bio Inc (Shiga, Japan). CCK-8 was purchased from DOJINDO LABORATORIES (Kumamoto, Japan). NHEK and the culture medium, Humedia KB2, including the additive agents, were purchased from Kurabo (Osaka, Japan). A receptor-cofactor assay system (RCAS) kit was purchased from Fujikura Kasei (Tokyo, Japan). TEST SKIN^TM^ LSE-high was purchased from Toyobo (Osaka, Japan).

### Preparation of sphingoid bases from glucosylceramide

4,8-Sphingadienine (d18:2) and 4-hydroxy-8-sphingenine (t18:1) were prepared by acidolysis of konjac glucosylceramide and column chromatography. Glucosylceramide (1.5 g) was incubated with 400 mL of methanol containing 1 N HCl at 70 °C for 18 h, which was followed by extraction with 100 mL hexane twice, to eliminate methyl esters of fatty acids. Sodium hydroxide solution (4 N) was used to neutralize the water phase. Chloroform and methanol were added to the mixture to attain a 1:1:1 (v:v:v) ratio of chloroform:methanol:water, which was followed by vigorous agitation. The chloroform phase was collected and concentrated *in vacuo*. The resulting residue was dissolved in chloroform and loaded onto a Chromatorex NH-DM1020 column (30 mm × 170 mm; Fuji Silysia Chemical, Kasugai, Japan) for column chromatography. Stepwise elution was performed with chloroform/methanol at ratios of 100:0 (150 mL), 90:10 (150 mL), 70:30 (150 mL), and 50:50 (300 mL). The eluents collected were analyzed by HPLC after solvent evaporation.

### HPLC analysis of sphingoid bases

The sphingoids prepared were analyzed by HPLC by using a Supersphere 100 RP-18(e) column (Merck, Japan), at 40°C. Isocratic elution was performed using methanol/distilled water/ammonium acetate in a ratio of 95:5:0.1 (v/v/w) as mobile phase. This was followed by detection using an evaporative light scattering detector (ELSD; Alltech, Deerfield, USA).

### Cell culture

NHEK was cultured using Humedia-KB2 medium with growth enhancers, viz., epidermal growth factor, insulin, hydrocortisone, bovine pituitary extract, and an antimicrobial agent. NHEK cells (10,000) were seeded in 24-well plates and cultured for 4 d; medium was exchanged every 2 d. On the fifth day, the medium was replaced with new medium supplemented with test materials, at the indicated concentrations: 4-sphingenine (5 μg/mL, 16.7 μM); 4,8-sphingadienine (5 μg/mL, 16.8 μM); 4-hydroxy-8-sphingenine (5 μg/mL, 15.8 μM); and glucosylceramide (13 μg/mL, 16.9 μM). After a 24 h cultivation, cells were used to assess gene expression.

TEST SKIN^TM^ LSE-high was cultured in the medium supplied by the manufacturer, and cultivation was performed according to the instruction manual. The medium was adjusted at the indicated concentration, 4,8-sphingadienine (2.5 μg/ml, 8.4 μM and 10 μg/ml,33.4 μM), 4-hydroxy-8-sphingenine (2.5 μg/ml, 7.9 μM and 10 μg/ml, 31.7 μM) and glucosylceramide (26 μg/ml, 33.9 μM), and the medium was exchanged 48 h after the start of cultivation. By 72 h after the start of culturing, the topmost keratinocytes were removed and analyzed.

### RNA extraction and real-time PCR

Total RNA was isolated from NHEK cell lysates 24 h after adding test samples, using Isogen reagent according to the manufacturer’s protocol. cDNA was synthesized from total RNA by using a PrimerScript RT reagent kit with an oligo-dT primer. Real-time quantitative RT-PCR analysis was performed using SYBR-Premix Ex-taq and automated sequence detection systems (StepOne; Applied Biosystems Japan Ltd, Tokyo, Japan). PCR cycling was performed using 2 conditions. Condition 1 involved 40 cycles, each consisting of 15 s at 95°C and 1 min at 60°C, and was used for serine palmitoyltransferase long chain (SPTLC) 2, dihydroceramide desaturase (DEGS)-1, ceramide synthase (CERS) 2–6, glucosylceramide synthase (GCS), β-glucocerebrosidase (β-GCS), the elongases of very long-chain fatty acids (ELOVL)-1, 4–7, peroxisome proliferator-activated receptor (PPAR)β/δ, γ amplification. Condition 2 involved 40 cycles, each consisting of 15 s at 95°C, 15 s at 60°C, followed by 1 min at 72°C, and was used for sphingomyelin synthase (SMS)-1,2 and sphingomyelinase (SMase). The sequences of the primers are shown in Table 1. The sequences of the primers were designed by the references [12-21], software and web site. 

**Table 1:** Primers for quantitative RT-PCR

**Gene**	**Primer sequence (Forward)**	**Primer sequence (Reverse)**	**Reference**
SPTLC2	CCAGACTGTCAGGAGCAACCATTA	CGTGTCCGAGGCTGACCATA	
CerS2	CCGATTACCTGCTGGAGTCAG	GGCGAAGACGATGAAGATGTTG	[12]
CerS3	ACATTCCACAAGGCAACCATTG	CTCTTGATTCCGCCGACTCC	[12]
CerS4	CTTCGTGGCGGTCATCCTG	TGTAACAGCAGCACCAGAGAG	[12]
CerS5	GCCATCGGAATCAGGAC	GCCAGCACTGTCGGATGT	[12]
CerS6	GGGATCTTAGCCTGGTTCTGG	GCCTCCTCCGTGTTCTTCAG	[12]
DEGS-1	GCGTTTGGCAGTTGCATTAA	CATTGTGGGCAATCTCATGAA	designed by Primer Express
SMS-1	GCCAGGACTTGATCAACCTAACC	CCATTGGCATGGCCGTTCTTG	[13]
SMS-2	CACCCAGTGGCTGTTTCTGA	TGCATTCCAGGCACAGGTAGA	[13]
aSMase	TGGCTCTATGAAGCGATGGC	TTGAGAGAGATGAGGCGGAGAC	[14]
β-GCS	GCTAGGCTCCTGGGATCGAG	GTTCAGGGCAAGGTTCCAGTC	[15]
GCS	GTTCGTCCTCTTCTTGGTGC	AGAAGAGAGACACCTGGGAGC	[16]
ELOVL-1	ATTCTCCTGACCTACGTGTACTT	TTCCGATTAGCCATGATGCGA	Primer Bank
ELOVL-4	CATGTGTATCATCACTGTACG	AAAGGAATTCAACTGGGCTC	[17]
ELOVL-5	TAACAGGAGTATGGGAAGGCA	ACCAGAGGACACGGATAATCTT	Primer Bank
ELOVL-6	TGCTGCCTTTATATTCGGTGG	CCTCAGTTCAAACTTTGCTCGTT	Primer Bank
ELOVL-7	TTCCATCATACCATCATGCC	CCCAATGCAGAAAGTCCATA	[18]
PPARβ/δ	ACAGCATGCACTTCCTTCCA	TCACATGCATGAACACCGTA	[19]
PPARγ	ATTCTGGCCCACCAACTTTG	TCCATTACGGAGAGATCCACG	[20]
GAPDH	TGCACCACCAACTGCTTAGC	GGCATGGACTGTGGTCATGAG	[21]

Primer sequences used in real-time quantitative RT-PCR in this study. All primers were used with SYBR-Green and their specificities checked using the melting curve method (60–95°C).

### Nuclear receptor-binding assays

Nuclear receptor-binding assays were performed using an RCAS kit according to the manufacturer’s protocol. All the results were normalized to the negative control (ETOH). GW1929, included in the kit, was used as the positive control.

### Ceramide extraction

The topmost keratinocyte layer was removed from the cultured TEST SKIN^TM^ LSE-high and cut into small pieces. These pieces were soaked in 2 mL of chloroform/methanol (1:2, v/v) and were left to stand at room temperature for 1 h to extract the ceramides. The liquid phase was transferred to a collection tube, and the residual keratinocytes were extracted in the same manner twice more, using chloroform/methanol/distilled water (1:2:0.5, v/v) and chloroform, and all extracts were pooled. To this was added 600μL of 2.5% KCl and 6 mL of distilled water; the samples were mixed well, followed by centrifugation at 900 × *g* at room temperature for 5 min. The lower phase was transferred to a new tube, and the upper layer was re-extracted with 4 mL of chloroform by vortexing the tube for 30 s, followed by a similar centrifugation step. The extract was dried at 50°C under a stream of nitrogen gas. The residue was dissolved in 100 μL chloroform/methanol (2:1, v:v) and was stored in a screw-top bottle at −20°C until use [22].

### HPTLC analysis of extracted lipids

The extracted lipids were analyzed by means of high-performance thin-layer chromatography (HPTLC) on an HPTLC plate (Merck, Tokyo, Japan); the following mobile phases and distances were used, in this order:

1. Chloroform, for 15 mm

2. Chloroform/acetone/methanol (76:8:16, v:v:v), for 10 mm

3. Chloroform/hexylacetate/acetone/methanol (86:1:10:4, v:v:v:v), for 70 mm

4. Chloroform/acetone/methanol (76:4:20, v:v:v), for 20 mm

5. Chloroform/diethylether/hexylacetate/ethylacetate/acetone/methanol (72:4:1:4:16:4, v:v:v:v:v:v), for 75 mm

6. Hexane/diethylether/ethylacetate (80:16:4, v:v:v), for 90 mm

After the solvent had evaporated, the HPTLC plate was sprayed with acetate/H_2_PO_4_/H_3_PO_4_/0.5% CuSO4 solution (5:1:1:95, v:v:v:v) and heated to 160 °C to visualize the separated lipids. The colored plate was immediately scanned using a densitometer.

### Statistical analysis

All the data are presented as mean ± SD values. Statistical significance was determined using Student’s *t*-test; *p* < 0.05 was considered to be statistically significant.

## Results

### Ceramide de novo synthesis-related gene expression

Purified glucosylceramide (≥99%) from konjac tuber was digested to sphingoids in the presence of hydrochloric acid. Table 2 shows the composition of sphingoids analyzed by GC-MS. The major sphingoid moieties of konjac glucosylceramide are 4,8-sphingadienine and 4-hydroxy-8-sphingenine. We examined the effects of the major sphingoids derived from konjac glucosylceramide on the expression of genes related to *de novo* synthesis of ceramide by using real-time quantitative PCR.

**Table 2:** Sphingoid composition of konjac glucosylceramide

**Sphigoid base**	**double bond**	**composition(%)**
t18:0	-	1.4
t18:1	C8-C9	40.2
d18:1	C8-C9	3.8
d18:1	C4-C5	0.6
d18:2	C4-C5,C8-C9	54.0

Sphingoid bases are presented in abbreviated style. d18:0 has 2 hydroxyl groups and 18 carbons, without any double bonds, whereas t18:1 has 3 hydroxyl groups, and 18 carbons, with 1 double bond.

First, we checked the toxicity of the sphingoids to the NHEK cells. CCK-8 was used to measure the cell viability after 24 h exposure of sphingoids. There were no differences between cells after sphingoids and vehicle treatment.

**Figure 2:**
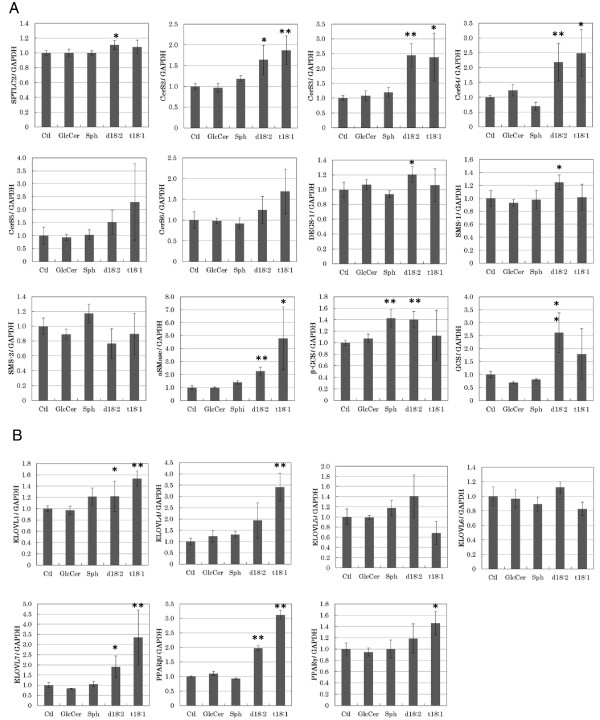
**Expression of genes involved in* de novo *synthesis of ceramides in sphingoid-treated NHEK cells.(A)** Expression of genes involved in *de novo* synthesis of ceramides. **(B)** Expression of ELOVL genes and PPAR genes. Real-time PCR was performed at 24 h after treatment of NHEK cells with sphingoids or glucosylceramide. Vehicle-treated (EtOH at a final concentration of 0.1%, which did not affect cell viability) NHEK cells were used as the control; gene expression was evaluated relative to these control cells. As test treatments, 5 μg/mL of 4-sphingenine (Sph), d18:2, or t18:1, or 13 μg/mL of glucosylceramide (GlcCer) was used. All the results are shown as mean ± SD (n = 4). *p < 0.05, **p < 0.01, significantly different from the level in the vehicle-treated control cells (Student’s *t*-test).

Then we used the assessed the expression of these genes in d18:2 and t18:1-treated NHEK cells. As shown in Fig. 2A, the expression of *SPTLC2*, *CerS3*, *DEGS-1*, *SMS-1*, *aSMase*, and *GCS* increased by 1.1-fold, 2.4-fold, 1.4-fold, 1.2-fold, 2.2-fold, and 2.6-fold, respectively, following exposure of the cells to 5 μg/mL d18:2. The expression of *CerS3*, *aSMase*, and *GCS* increased by 2.5-fold, 4.7-fold, and 1.3-fold, respectively, in the presence of 5 μg/mL t18:1.

Second, we tested the expression of *ELOVL-1*, *ELOVL-4*, *ELOVL-5*, *ELOVL-6*, and *ELOVL-7*. As shown in Fig. 2B, the expression of *ELOVL-1*, *ELOVL-4*, and *ELOVL-7* increased by 1.2-fold, 1.9-fold, and 1.9-fold, respectively, in the presence of 5 μg/mL d18:2. Moreover, in the presence of 5 μg/mL t18:1, the expression of these 3 genes increased by 1.5-fold, 3.4-fold, and 3.3-fold, respectively.

We also tested the expression of *PPARβ/δ,* and *PPARγ*, which are known to promote the differentiation of keratinocytes. As shown in Fig. 2BZ, expression of *PPARβ/δ* in the presence of 5 μg/mL d18:2 or t18:1 increased by 2.0-fold and 3.0-fold, respectively. *PPARγ* expression increased by 1.4-fold in the presence of 5 μg/mL t18:1. In contrast, neither glucosylceramide nor 4-sphingenine had any marked effects on the expression of these genes.

### PPARγ ligand

**Figure 3:**
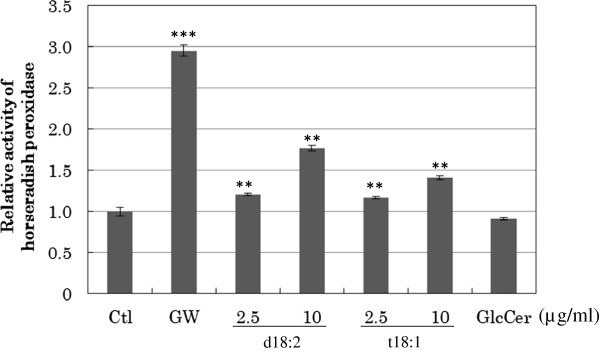
**Activation of PPARγ by sphingoids.** The ability of 2.5 and 10 μg/mL of sphingoids or 26 μg/mL glucosylceramide to bind and activate PPARγ was measured using a receptor cofactor assay system. As positive control, 8 mM of GW1929 was used. Horseradish peroxidase activity was normalized to the absorption at 450 nm. All the results are shown as mean ± SD values (n = 3). **p < 0.01, ***p < 0.001, significantly different from the levels in vehicle-treated control cells.

The ability of d18:2 and t18:1 to act as ligands of PPARγ was assessed using nuclear receptor assays. As shown in Fig. 3, no binding of glucosylceramide to PPARγ was observed. In contrast, d18:2 and t18:1 showed efficient binding to PPARγ. These results suggested that d18:2 and t18:1 are ligands of PPARγ and thus participate in positive feedback regulation of PPARγ activity, including induction of differentiation in keratinocytes.

### Ceramide sysnthesis in 3-dimensional human skin model

**Figure 4:**
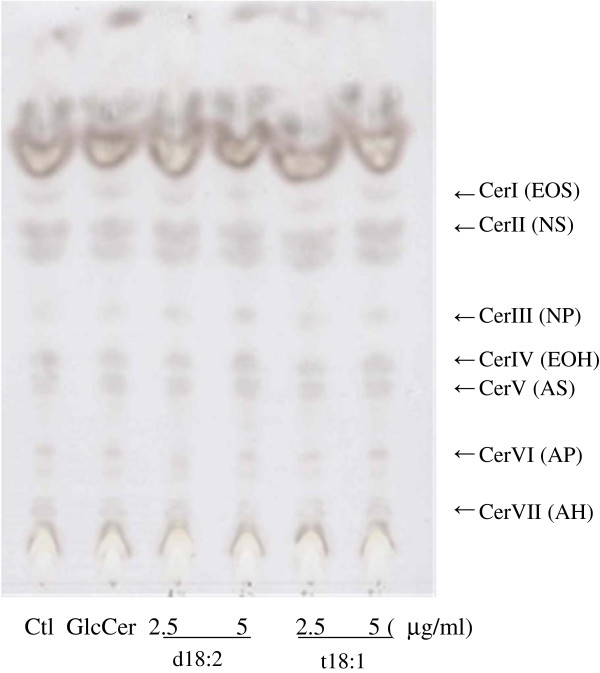
**HPTLC of ceramides extracted from a 3-dimensional human skin model treated with sphingoids.** Two wells containing 3-dimensional human skin model samples were pooled per sample. The weight of the keratinocyte layer was measured soon after the removal of this layer from the model; the values for all samples were subsequently adjusted to this weight. The same amount of lipids extracted from the keratinocyte layer of all samples was loaded onto the HPTLC plate. To visualize the ceramides, the HPTLC plate was treated with acetate/H_2_PO_4_/H_3_PO_4_/0.5% CuSO_4_ solution and heated. Abbreviations of Cer I to VII represent Ceramide I to VII, these are classical names base on rate of flow in TLC. Recent names based on the structure, the combination of fatty acid and sphingoids, were shown in the parenthesis. A = α-hydroxy fatty acid, EO = Esterified ω-hydroxy fatty acid, N = Non-hydroxy fatty acid, P = Phytosphingosine, S = Sphingosine, H = 6-Hydroxy sphingosine.

To assess the upregulation of ceramide production by d18:2 and t18:1, we used a reconstituted human skin model, TEST SKIN^TM^ LSE-high. As shown in Fig. 4, we identified 7 ceramide spots [23]; d18:2 treatments of the skin model increased the intensities of ceramide III (NP) spots, while t18:1 treatments increased the intensities of ceramide I (EOS), II (NS), and III (NP), compared to vehicle (ethanol) and glucosylceramide treatments. These results are summarized in Table 3, in which the digitized intensities of the spots are recorded. These results indicate that d18:2 and t18:1 induce ceramide production in a human skin model. 

**Table 3:** Levels of ceramide in a 3-dimensional human skin model treated with sphingoids

	**Ctl**	**GlcCer**	**d18:2 2.5 μg/mL**	**d18:2 5 μg/mL**	**t18:1 2.5 μg/mL**	**t18:1 5 μg/mL**
Cer I (EOS)	1.00	0.82	0.87	0.98	1.10	1.27
Cer II (NS)	1.00	0.78	0.92	0.91	1.18	1.20
Cer III (NP)	1.00	1.24	1.19	1.90	1.65	1.83

A 3-dimensional human skin model, TEST SKIN^TM^, was cultured in medium containing sphingoids or glucosylceramide for 72 h. Ceramides were then extracted from the topmost keratinocyte layer and analyzed by HPTLC. The levels of ceramides were assessed by densitometry; results were evaluated relative to the densities in vehicle-treated control samples.

## Discussion

Although previous reports have demonstrated that dietary glucosylceramide can decrease TEWL from human skin [24], the mechanism was unclear. Ueda *et al*. reported that orally administrated ceramide was distributed to the dermis after intestinal absorption, followed by transfer from the dermis to the epidermis [25]. Ishikawa *et al*. also showed that dietary glucosylceramide was degraded into sphingoids, which were absorbed through the intestinal epithelial cells of rats and were subsequently also found in the lymph fluid in these rats [11]. Another study showed that 4-hydroxysphinganine could activate the PPARs [26] that are the key regulators of keratinocyte differentiation [27].

Keratinocyte differentiation is closely related to internal ceramide synthesis [28]. It is unlikely that orally ingested glucosylceramide would specifically localize to the skin because the amount of ingested glucosylceramide is simply too little to enhance ceramide levels in the skin. We hypothesized that sphingoids are the most likely effectors of ceramide production in the skin. In the present study, we used d18:2 and t18:1 as candidates because they were the major constituents of konjac glucosylceramide, as shown in Table 2. Daily oral intake of 1.8 mg konjac glucosylceramide for 28 days had improved transepidermal water loss (TEWL) of the skin in a human trial [10]. Previous report [26] showed that phytoceramide activated PPARs at the concentration of above 10 μM. According this report, it was 10 μM that we assumed the enough concentration for the activation of ceramide *de novo* synthesis. In this study (*in vitro*), the concentration was adopted from 8.4 (2.5 μg/ml) to 33.4.μM (10 μg/ml) for sphingoids and glucosylceramide. As a result, the upregulation of ceramide *de novo* synthesis, the activation of PPARγ and the induction of ceramides in 3-dimensional human skin model have been cofirmed.

Real-time quantitative RT-PCR was performed using NHEK cells that had been treated with sphingoids and glucosylceramide. We tested the effects of d18:2 and t18:1 on the expression of genes related to *de novo* ceramide synthesis, including *SPTLC2DEGS-1*, and *CerS2-b*[29,30], the *ELOVL* family, and *PPARs*. Glucosylceramide and 4-sphingenine had no noticeable effects on the expression of these genes; in contrast, d18:2 and t18:1 could activate the expression of these genes. Among the products of the genes activated, CerS3 catalyzes the production of epidermal ceramides [7], and CerS2 regulates ELOVL-1–mediated synthesis of C22-CoA from C18 via C20, which is an essential step in the production of C24 ceramide [31]. Our data therefore indicated the possibility of induction of keratinocyte differentiation and ceramide production as a result of treatment with d18:2 and t18:1.

We next attempted to assess the activatory effects of d18:2 and t18:1 on PPARγ by using *in vitro* binding assays. We found that d18:2 and t18:1 could activate PPARγ, whilst glucosylceramide could not. The results of our gene expression studies and binding assays indicated that d18:2 and t18:1 promote the differentiation of keratinocytes and stimulate endogenous ceramide production.

When we investigated the effects of these compounds on TEST SKIN^TM^ LSE-high, we observed that ceramides I, II, and III were induced by d18:2 and t18:1 treatment. This indicated that d18:2 and t18:1 promoted not only the induction of gene expression but also the production of ceramides in human skin.

As mentioned earlier, various reactions were induced by d18:2 and t18:1 but not by 4-sphingenine. The major structural difference between the active sphingoids and 4-sphingenine is a C8–C9 double bond. A previous report demonstrated that 4-hydroxysphinganine (t18:0) could act as a ligand for PPARs [26,32]. This is the first report that t18:1 and d18:2 are the agonists for PPARγ. As 4-sphingenine (d18:1) did not act as PPARγ agonist, some functional molecular structure, for example 4-hydroxy (t18:0 and t18:1) or C8 double bond (t18:1 and d18:2), might be needed to activate PPARs.

Nine species of sphingoids are commonly found in plants [33], and many sources of glucosylceramide other than konjac tuber exist, e.g., rice bran, corn, apple, and sugar beet pulp. These glucosylceramides also contain d18:2 and t18:1, but these sphingoids are not as abundant as in konjac, suggesting that konjac is the most effective source of material for improving skin ceramide production.

Water retention of the skin decreases because of aging, UV exposure, and diseases such as atopic dermatitis [7]. These factors cause the depletion of skin ceramides and a reduction in hyaluronic acid, and lead to inflammation and wrinkling of the skin. In contrast, dietary glucosylceramides can improve the condition of the skin [34,35]. Because the amount of glucosylceramide ingestion is so limited, it is believed that improvement of the skin is not due to the direct localization of glucosylceramide to the skin. In this study, we have shown that at least part of the mechanism involves a triggering of the ceramide synthesis pathway by d18:2 and t18:1. Further studies may help elucidate the complete mechanism underlying efficient improvement of skin conditions through dietary glucosylceramides, thereby contributing to human health and wellbeing.

## Competing interests

The authors declare that they have no competing interests.

## Authors’ contributions

YS participated in the design of the study, carried out analysis and interpretation of data and drated the manuscript. KK and Kenji M carried out processing of glucosylceramide. Katsuyuki M participated in the design of the study, contributed to the interpretation of data and revised the manuscript. SM and YI participated in the design of the study and contributed to the interpretation of data. All authors read and approved the final manuscript.
